# Clinical Relevance of Routine Follow-Up Computed Tomography in Dogs with Presumed Discospondylitis: A Retrospective Study

**DOI:** 10.3390/vetsci13101057

**Published:** 2026-10-08

**Authors:** Eva Ingelaere, Laurent Blond, Robin Cavalerie, Antoine Dunié-Mérigot, Clément Baudin-Tréhiou

**Affiliations:** 1 Department of Diagnostic Imaging, Centre Hospitalier Vétérinaire Languedocia, 34080 Montpellier, France; 2 Department of Neurology, Centre Hospitalier Vétérinaire Languedocia, 34080 Montpellier, France; 3 Department of Surgery, Centre Hospitalier Vétérinaire Languedocia, 34080 Montpellier, France

**Keywords:** spinal infection, vertebral endplate, disease monitoring, diagnostic imaging, longitudinal imaging

## Abstract

Computed tomography (CT) findings of discospondylitis in dogs are well described, but limited information is available regarding the clinical value of follow-up CT in veterinary medicine. This retrospective study evaluated CT changes and their associations with clinical outcome in 32 dogs with suspected discospondylitis. Initial and follow-up CT examinations were independently reviewed by two board-certified veterinary radiologists. Disagreements were resolved by agreement between reviewers, and changes in predefined CT abnormalities were combined into a CT Evolution Score. Interobserver agreement for individual CT features ranged from fair to substantial, while the CT Evolution Score showed moderate reliability (ICC = 0.72). Dogs with a good clinical outcome showed more favorable changes in paravertebral soft tissue abnormalities (*p* < 0.001) and presumptive spinal epidural empyema (*p* = 0.002), whereas osseous changes did not differ significantly. The CT Evolution Score was higher in dogs with a good outcome (*p* = 0.010), associated with lower odds of a poor outcome (OR, 0.40; 95% CI, 0.17–0.91; *p* = 0.029), and showed good discriminatory ability (AUC = 0.87). Follow-up CT may provide complementary information, particularly in dogs with incomplete or unfavorable clinical improvement.

## Simple Summary

Discospondylitis is an infection affecting the disc and adjacent bones of the spine in dogs. Computed tomography is useful for diagnosing this condition, but it is unclear whether repeating this examination during treatment provides useful information about recovery. This study aimed to describe changes seen on follow-up computed tomography examinations and determine whether these changes were related to the dogs’ clinical recovery. Initial and follow-up examinations from 32 dogs with suspected discospondylitis were reviewed. Dogs that recovered well showed greater improvement in abnormalities affecting the soft tissues surrounding the spine and suspected infection within the spinal canal, whereas changes affecting the bones were not clearly related to clinical recovery. Overall improvement on computed tomography was associated with a better clinical outcome. These findings suggest that follow-up computed tomography may provide useful additional information when monitoring affected dogs, particularly when their clinical improvement is incomplete or unsatisfactory.

## 1. Introduction

Discospondylitis is an infectious disease affecting one or more intervertebral disc (IVD) spaces and adjacent vertebral endplates, which may extend to the vertebral bodies, surrounding soft tissues, or vertebral canal [1,2]. As clinical signs are often variable and nonspecific, diagnosis is typically based on imaging characteristics [1,2,3,4]. Although magnetic resonance imaging (MRI) provides superior soft tissue contrast for assessing paravertebral and vertebral canal involvement [1,3,4,5,6], computed tomography (CT) provides detailed assessment of the osseous changes associated with discospondylitis, including vertebral endplate lysis and irregularity, sclerosis, and periosteal proliferation, and may also identify associated paravertebral or epidural abnormalities [3,7].

Treatment of discospondylitis primarily relies on prolonged antimicrobial therapy, although surgical intervention may be indicated in selected cases [1,2,8]. Broad-spectrum antimicrobials, such as cephalexin and amoxicillin–clavulanic acid, are among the most commonly prescribed antimicrobials, although antimicrobial selection should ideally be guided by bacterial culture and susceptibility testing [1,8,9]. The optimal duration of antimicrobial treatment remains uncertain, and premature discontinuation may result in recurrence [1,2,8]. Follow-up imaging may therefore complement clinical assessment when monitoring treatment response and determining when treatment can be discontinued.

In dogs, follow-up radiographic changes have been described in a small cohort of dogs with successful clinical recovery, with radiographic deterioration continuing for several weeks despite early clinical improvement before subsequent radiographic resolution [10]. More recently, follow-up magnetic resonance imaging (MRI) has also been investigated in dogs, with persistent or progressive MRI abnormalities reported in some clinically improving patients, suggesting that MRI findings may not necessarily reflect clinical recovery [11]. In human patients with discospondylitis, follow-up CT has been reported to demonstrate progressive resolution of inflammatory changes and increasing osseous consolidation during treatment [12], although imaging abnormalities may persist or progress despite clinical improvement [13,14]. However, the evolution of CT abnormalities during routine follow-up of canine discospondylitis and their relationship with clinical improvement remain poorly described.

The aim of this retrospective study was therefore to evaluate changes in CT findings during follow-up of dogs with suspected discospondylitis and to assess their relationship with clinical outcome. We hypothesized that follow-up CT would demonstrate progressive resolution of inflammatory soft tissue changes, together with a decrease or resolution of vertebral lysis and increasing vertebral sclerosis and osseous bridging during treatment, consistent with patterns of recovery previously described in dogs on follow-up radiographs and in human patients with discospondylitis. We further hypothesized that these CT changes would be associated with clinical outcome.

## 2. Materials and Methods

### 2.1. Study Design and Case Selection

This retrospective observational cohort study of client-owned dogs was conducted at a single referral veterinary center (Centre Hospitalier Vétérinaire Languedocia, Montpellier, Occitanie, France). Electronic medical records were searched using the keyword “discospondylitis” to identify dogs that underwent CT examination and were presumptively diagnosed with discospondylitis between 1 April 2016 and 1 May 2026. Dogs were eligible for inclusion if they had (1) a presumptive diagnosis of discospondylitis based on compatible clinical signs and CT abnormalities, (2) an initial CT examination of the affected spinal region, and (3) at least one follow-up CT examination of the affected region performed during antimicrobial treatment. Dogs undergoing surgical treatment were eligible for inclusion. Dogs were excluded if required clinical data or CT images were missing or incomplete.

All CT examinations were reviewed and approved for inclusion by an imaging team comprising two board-certified veterinary radiologists (LB and CBT) and a resident in diagnostic imaging (EI). For case selection, the initial CT examination was reviewed, blinded to the subsequent clinical outcome and the follow-up CT findings, to assess whether the findings were consistent with a presumptive diagnosis of discospondylitis, based on previously described CT findings in dogs [1,3,15,16,17]. They included endplate involvement, endplate erosion or osteolysis, vertebral body osteolysis, periosteal proliferation, sclerosis of the vertebral body or endplates, and abnormalities of the IVDS, as well as paravertebral and epidural soft tissue abnormalities when present. Final inclusion decisions were made by agreement among the three members of the imaging team.

### 2.2. Data Collection and Analysis

Among dogs included in the final study population, data were retrieved from electronic medical records by the diagnostic imaging resident (EI).

#### 2.2.1. Initial Clinical Data

Initial clinical data collected included patient signalment (age, breed, sex, and body weight), duration of clinical signs prior to referral, potential predisposing factors (e.g., previous surgical procedures and the time interval between surgery and the onset of discospondylitis), and presence of pyrexia (>39.2 °C). Onset of clinical signs was classified as acute (<7 days), subacute (7–30 days), or chronic (>30 days). Neurological status at admission was graded using a modified Frankel Scale (MFS) as follows: grade 0, neurologically normal; grade 1, spinal pain without neurological deficits; grade 2, ambulatory paraparesis and/or ataxia; grade 3, non-ambulatory paraparesis; grade 4, paraplegia with preserved nociception; and grade 5, paraplegia with absent nociception. When available, C-Reactive Protein (CRP) concentration was collected. Microbiological results were also recorded, including aerobic and anaerobic bacterial culture results and identified pathogens, together with the source of the sample (urine, blood, direct IVD sampling by ultrasound- or CT-guided percutaneous aspiration, intraoperative sampling, or other sources). Cytological and histopathological findings were also recorded when available. Urine samples were collected before contrast medium injection. *Brucella canis* serology results were recorded separately. Treatment administered for discospondylitis was recorded and classified as medical when antimicrobial therapy alone or in combination with anti-inflammatory and/or analgesic treatment, or as surgical when surgery preceded antimicrobial therapy.

#### 2.2.2. Follow-Up Clinical Data and Outcome

Follow-up clinical data included the clinical and neurological evolution compared with the initial presentation, as well as changes in CRP concentrations, when available. For dogs with serial CRP measurements, the change in CRP concentration was assessed between consecutive measurements and categorized as decreased, unchanged, or increased.

Outcome was classified as good when neurological signs improved or resolved, based on changes in the modified Frankel Scale (MFS) and clinical assessment, and as poor in cases of no clinical improvement, relapse, or euthanasia related to discospondylitis.

The time interval between the initial and follow-up CT examinations, or between two different follow-up CT examinations, was also recorded.

#### 2.2.3. Initial and Control Imaging Data Acquisition

All initial and follow-up CT examinations were performed under general anesthesia using a 16-slice helical multidetector CT scanner (Optima 16, General Electric, Chicago, IL, USA). Acquisition parameters, contrast timing, and patient positioning varied according to the imaging protocol used, with the spinal protocol being the most frequently applied. This protocol consisted of transverse pre- and post-contrast medium acquisitions of the spine using a soft tissue reconstruction algorithm. The post-contrast medium acquisition was obtained approximately 40 s after the beginning of intravenous administration of iodinated contrast medium (Omnipaque, GE Healthcare, Oslo, Norway; 350 mg I/mL), corresponding to the venous phase. Acquisition parameters included a slice thickness of 1.25 mm, a pitch factor of 0.562, a rotation time of 0.7 s, a tube voltage of 120 kVp, and a tube current ranging from 150 to 300 mAs. Dogs were positioned in dorsal recumbency. Bone algorithm reconstructions with a slice thickness of 0.625 mm were also routinely available.

#### 2.2.4. CT Images Evaluation and Grading Criteria

Images were reviewed using a dedicated DICOM viewing software (Synapse PACS, version 7.3.100; Healthcare Americas Corporation, Lexington, MA, USA). Initial and follow-up examinations were independently reviewed in a randomized order by the two board-certified veterinary radiologists (LB and CBT), who assessed the predefined CT abnormalities and were blinded to each other’s assessments and to the clinical outcome. Discrepancies between readers were resolved by mutual agreement, and the resulting consensus assessments were used for all subsequent analyses.

Initial and control CT examinations were systematically assessed for lesion characteristics, including the number and location of affected IVDS, vertebral subluxation, and associated paravertebral or epidural abnormalities. Paravertebral soft tissue thickening was recorded as a qualitative imaging finding characterized by a relatively well-delineated soft tissue expansion ventral or lateral to the affected IVD space, associated with contrast enhancement compared with adjacent normal paravertebral musculature. Presumptive spinal epidural empyema (SEE) was defined as a loss of normal epidural fat signal, replaced by heterogeneous soft tissue-attenuating material, often ring-enhancing after contrast medium injection [7].

For dogs with more than one follow-up CT examination, lesion evolution was assessed sequentially, with each follow-up examination compared with the preceding CT examination.

Changes between consecutive CT examinations were categorized as unfavorable, unchanged, or favorable. The classification of changes as favorable or unfavorable and the CT Evaluation Score were based on patterns of imaging evolution previously described in the human and veterinary literature [6,10,18,19]. As no established weighting of individual CT features is available, all features included in the CT Evolution Score were assigned equal weight. For destructive or inflammatory abnormalities, including osteolysis and paravertebral or epidural soft tissue changes, a qualitative decrease in extent or severity was considered favorable, whereas an increase in extent or severity was considered unfavorable. Conversely, a qualitative increase in reparative osseous changes, including IVDS narrowing, periosteal proliferation and/or bridging, was considered favorable. Vertebral sclerosis was assessed separately and was not incorporated into the CT Evolution Score because its presence may reflect either reparative bone remodeling or an ongoing osseous response and therefore could not be unambiguously classified as favorable or unfavorable. A CT Evolution Score was calculated by assigning −1 point for an unfavorable or newly developed abnormality, 0 for no change, and +1 point for a favorable change or complete resolution, as described in Table 1.

Individual abnormality scores were summed to obtain a CT Evolution Score for each affected IVDS. A positive CT Evolution Score indicated a predominance of favorable changes among the assessed CT abnormalities, whereas a negative score indicated a predominance of unfavorable changes. A score of zero indicated no net change across the assessed CT abnormalities. In dogs with multifocal discospondylitis, each affected IVDS was assessed separately and assigned an individual CT Evolution Score. For descriptive analyses, including the analysis of individual CT features, each affected IVDS was considered separately, without averaging across affected IVDS. For statistical analyses evaluating associations with clinical outcome, only the first follow-up examination was included to ensure a single observation per dog and avoid non-independence from repeated examinations. Values from multiple affected IVDS were averaged to obtain a single value per dog.

#### 2.2.5. Statistical Analysis

Data analysis was performed using the open-source software jamovi (version 2.7.38; The jamovi Project). Categorical variables were summarized as counts and percentages. Clinical outcome was considered a binary variable (good or poor), individual CT feature evolution scores were treated as numerical variables derived from ordinal scores, and the CT Evolution Score was treated as a quantitative variable. The normality of quantitative variables was assessed using the Shapiro–Wilk test.

Interobserver agreement between the 2 board-certified veterinary radiologists (LB and CBT) was assessed using linearly weighted Cohen’s kappa (κ) for individual ordinal CT features. Gwet’s agreement coefficients were additionally calculated to provide a more robust estimate of agreement in the presence of imbalanced category distributions. Interobserver reliability for the CT Evolution Score was assessed using a two-way random-effects, single-measure intraclass correlation coefficient (ICC). Agreement coefficients were interpreted as follows: <0, poor; 0.00–0.20, slight; 0.21–0.40, fair; 0.41–0.60, moderate; 0.61–0.80, substantial; and 0.81–1.00, almost perfect agreement [20]. ICC values were interpreted as poor (<0.50), moderate (0.50–0.75), good (0.75–0.90), or excellent (>0.90) reliability [21].

Differences in the CT Evolution Score and individual CT features between dogs with good and poor clinical outcomes were assessed using the Mann–Whitney U test. Binary logistic regression was used to evaluate the association between CT Evolution Score and clinical outcome, with CT Evolution Score included as the sole predictor, with results expressed as an odds ratio (OR) with a 95% confidence interval (CI). Receiver operating characteristic (ROC) curve analysis was performed to assess the discriminatory ability of the CT Evolution Score for clinical outcome. The area under the curve (AUC) with 95% CI was calculated, and the optimal cut-off was determined using Youden’s index. A *p*-value < 0.05 was considered statistically significant.

## 3. Results

### 3.1. Initial Clinical Findings

A total of 81 dogs with a presumptive diagnosis of discospondylitis were identified from the electronic medical records. Of these, 39 dogs had both an initial and at least one follow-up CT examination. Seven dogs were excluded because of missing or incomplete required data, resulting in a final study population of 32 dogs. Demographic and clinical data of the population of dogs included in this study are summarized in Table 2.

Four dogs had prior surgeries, including dorsal hemilaminectomy, total hip replacement, Double Pelvic Osteotomy (DPO), and multiple pelvic procedures for bilateral sacroiliac disjunction with acetabular fracture. The interval between surgery and the onset of clinical signs of discospondylitis ranged from 10 days to 2 months. All pelvis surgeries involved implants. Nine dogs received antibiotics prior to referral, including amoxicillin–clavulanic acid (*n* = 3), cephalexin (*n* = 2), marbofloxacin (*n* = 1), and doxycycline (*n* = 1). In the remaining two dogs, previous antimicrobial treatment was reported, but the specific antimicrobial administered was not documented.

Spinal hyperesthesia was the most common clinical sign, observed in 21/32 cases (65.6%), while pyrexia was observed only in 7/32 cases (21.9%). Thirteen dogs (40.6%) had additional neurological symptoms at admission: 10 dogs had ambulatory tetraparesis or paraparesis (MFS grade 2), with or without ataxia and/or proprioceptive deficits, two had non-ambulatory tetraparesis or paraparesis (MFS grade 3), and one had paraplegia with preserved nociception (MFS grade 4). No dog presented with paraplegia associated with loss of nociception (MFS grade 5). Clinical signs had a predominantly subacute onset (18/32 dogs, 56.2%), followed by acute onset in 7/32 dogs (21.9%) and chronic onset in 7/32 dogs (21.9%).

CRP concentrations were available for 17/32 dogs, of which 13/17 (76.5%) had increased concentrations and 4/17 (23.5%) had concentrations within the reference interval.

Samples for bacteriological culture were obtained from different sites in 29/32 dogs (90.6%). Urine culture was performed in 19/32 dogs (59.4%) and was positive in 5/19 cases (26.3%). Direct IVD sampling was performed in 23/32 dogs (71.9%), including CT-guided aspiration in 11/23 (47.8%), ultrasound-guided aspiration in 9/23 (39.1%), and intraoperative sampling in 3/23 (13.1%), with one dog undergoing both CT-guided and intraoperative sampling. In one additional dog, the guidance method used for direct IVD sampling was not documented. Positive microbiological results were obtained in 10/23 dogs (43.5%). Blood culture was performed in one dog and was negative. Additional bacteriological samples were obtained from a prostatic aspirate, a surgically removed implant (unrelated to direct IVD sampling), and a joint aspirate. The prostatic culture was negative, whereas the other two samples yielded positive results. Detailed microbiological results are presented in Table 3.

Across all positive cultures, *Staphylococcus* spp. were the most frequently identified bacterial group, accounting for 9/17 positive cultures (52.9%). Concordant IVD and urine cultures were observed in 1/5 dogs with positive urine cultures. One additional dog had concordant IVD and surgical material cultures, whereas the joint aspirate yielded *Enterobacter cloacae* in a dog with a negative IVD culture.

Serological testing for *Brucella canis* was performed in 9/32 dogs, and all results were negative.

Cytological examination was performed in 8/32 dogs (25%) and revealed inflammatory changes in 2/8 dogs, while the remaining 6 samples were non-diagnostic. Histopathological examination was not performed in any dog.

### 3.2. Initial CT Findings

The site of infection was limited to a single IVDS in 25/32 dogs (78.1%), whereas multifocal discospondylitis was identified in 7/32 dogs (21.9%). Lesions were distributed across the cervical (2 sites), cervicothoracic (2), thoracic (15), thoracolumbar (1), lumbar (7), and lumbosacral (13) regions. Because some dogs had multifocal disease, individual dogs could contribute lesions to more than one spinal region, resulting in a total number of affected regions greater than the number of dogs.

Osteolysis of the vertebral endplates with or without vertebral body involvement was identified in 29/32 dogs (90.6%), IVDS narrowing in 20/32 (62.5%), and ventral periosteal proliferation and/or spondylosis in 31/32 (96.9%). Sclerosis of the vertebral endplates was additionally identified in 22/32 dogs (68.8%). In addition to these typical osseous changes, paravertebral soft tissue thickening was observed in 28/32 dogs (87.5%), with mild to marked heterogeneous contrast enhancement in most cases, presumptive SEE was identified in 17/32 dogs (53.1%), and vertebral subluxation was observed in two dogs (6.3%).

### 3.3. Clinical Evolution, Treatment, and Outcome

The median interval between the initial and first follow-up CT examinations was 2 months (range, 1–6 months). Five dogs underwent multiple follow-up CT examinations, with intervals between consecutive examinations ranging from 10 days to 6 months. Medical treatment alone was administered in 26/32 dogs (81.2%), whereas 6/32 dogs (18.8%) underwent both medical and surgical treatment. Amoxicillin–clavulanate was the most frequently administered antimicrobial, used in 25/32 dogs (78.1%), as most isolated pathogens were susceptible to this drug. Surgical interventions consisted of placement of a gentamicin-impregnated sponge within the affected IVDS in one dog following antimicrobial susceptibility testing, vertebral stabilization due to vertebral subluxation in one dog, surgical management of a sublumbar abscess secondary to a migrating vegetal foreign body in one dog, and surgical decompression of presumed compressive SEE in 3 dogs. The other dog with vertebral subluxation was managed conservatively. Antimicrobial treatment was modified because of antimicrobial resistance in 3 dogs, including the dog that underwent placement of a gentamicin-impregnated sponge.

At the first follow-up examination, 27/32 dogs (84.4%) had a good clinical outcome, whereas 5/32 (15.6%) had a poor outcome, including one dog that was euthanized because of lack of clinical improvement despite medical treatment. Among dogs with multiple follow-up examinations, one experienced clinical relapse after discontinuation of treatment, followed by clinical improvement after antimicrobial treatment was reinitiated. Another dog showed clinical deterioration and was subsequently euthanized because of lack of clinical improvement despite placement of a gentamicin-impregnated sponge, whereas the remaining 3 dogs continued to improve clinically.

Follow-up CRP concentrations were available for 17 dogs. In four dogs, CRP evolution could not be assessed because initial CRP concentrations were unavailable. Among these four dogs, follow-up CRP concentrations were increased in three, including two dogs with good clinical improvement and one with clinical deterioration, and were within the reference interval in one dog with resolution of clinical signs. Of the remaining 13 dogs, CRP concentrations normalized at follow-up in seven dogs, all of which showed good clinical improvement. CRP concentrations increased in two dogs, concurrent with clinical deterioration. In one of these, CRP subsequently normalized at the 6-month follow-up, whereas in the other, CRP concentrations decreased substantially at 3 months but remained above the reference interval. CRP concentrations also increased in 3 dogs despite good clinical improvement, with subsequent normalization in one dog at 7 months. One additional dog had CRP concentrations within the reference interval at both the initial and follow-up examinations and showed good clinical improvement.

### 3.4. Follow-Up CT Findings and CT Evolution

On first follow-up CT examinations, the evolution of CT abnormalities differed between clinical outcome groups. Endplate osteolysis, periosteal proliferation and/or spondylosis, and IVDS narrowing tended to increase or remain unchanged, particularly in dogs with a good outcome. In contrast, paravertebral soft tissue abnormalities and presumptive SEE more often decreased or resolved in dogs with a good outcome, whereas these findings were more frequently unchanged or increased in dogs with a poor outcome. Vertebral sclerosis showed a similar distribution between outcome groups, with approximately half of evaluated discospondylitis sites showing increased sclerosis. New discospondylitis foci developed in 2 dogs with a good outcome and 3 with a poor outcome at the first follow-up examination. Percentages were calculated according to the number of evaluated discospondylitis sites rather than the number of dogs, as individual dogs could have multiple affected sites. Changes in CT findings between the initial and first follow-up examinations for each individual IVDS and the corresponding percentages according to outcome are summarized in Figure 1. Complete osseous bridging of the affected IVDS was observed in 6/32 dogs (18.8%). All 6 dogs had a positive CT Evolution Score and a good clinical outcome.

At the second follow-up CT, involving 5 dogs, osteolysis and periosteal proliferation/spondylosis improved in 2/5 and 3/5 dogs, respectively, while IVDS narrowing and sclerosis remained unchanged in all dogs. Paravertebral soft tissue abnormalities improved in 2/5 dogs and worsened in one, whereas SEE improved in one and worsened in one. One new discospondylitis focus was identified.

At the third follow-up CT, involving 2 dogs, osteolysis improved in one dog and worsened in the other, while periosteal proliferation/spondylosis improved in both, including complete bridging in one. IVDS narrowing increased in one dog, and paravertebral abnormalities improved in both. SEE improved in one dog and remained unchanged in the other. No new focus was identified, while sclerosis increased in one dog and remained unchanged in the other.

### 3.5. Associations Between CT Evolution and Clinical Outcome

Dogs with a good outcome showed significantly more favorable changes in paravertebral soft tissues, presumptive SEE, and CT Evolution Score compared with dogs with a poor outcome (*p* < 0.05). No significant differences between outcome groups were observed for osteolysis, spondylosis, or IVDS narrowing (*p* = 0.493, 0.689, and 0.641, respectively). A higher CT Evolution Score was associated with lower odds of a poor clinical outcome (OR, 0.40; 95% CI, 0.17–0.91; *p* = 0.029). These results are summarized in Table 4.

ROC analysis showed an AUC of 0.87 (95% CI, 0.68–1.00; *p* < 0.001) for the CT Evolution Score in discriminating between good and poor clinical outcomes. The optimal cut-off identified by Youden’s index was −0.17, with 96.3% sensitivity and 60.0% specificity for identifying a good clinical outcome. Representative examples of CT changes between initial and follow-up examinations are shown in Figure 2 and Figure 3.

Interobserver agreement for individual CT features ranged from fair to substantial (linearly weighted Cohen’s κ = 0.382–0.701). For IVDS narrowing, Gwet’s AC2 indicated substantial agreement (0.728; 95% CI, 0.606–0.849). Interobserver reliability for the CT Evolution Score was moderate (ICC [2, 1] = 0.721; 95% CI, 0.557–0.831), as shown in Table 5.

## 4. Discussion

Although radiographic and MRI follow-up assessments of dogs with presumed discospondylitis have been previously described in the veterinary literature [10,11], and sequential CT findings have been reported in a single case report [22], to the authors’ knowledge, this is the first study to evaluate the clinical relevance of CT findings during follow-up in a population of dogs with suspected discospondylitis. Our results indicate that dogs with a good clinical outcome showed significantly greater improvement in paravertebral soft tissue and presumptive epidural changes, as well as higher CT Evolution Scores, whereas changes in osteolysis, spondylosis, and IVDS narrowing were not associated with outcome. In addition, higher CT Evolution Scores were associated with lower odds of a poor clinical outcome and showed good discriminatory ability for clinical outcome (AUC = 0.87), with moderate interobserver reliability (ICC = 0.72), supporting the potential value of evaluating CT changes collectively rather than relying on individual osseous features. These findings should, however, be considered exploratory.

Typical CT findings in discospondylitis include vertebral endplate osteolysis, with or without involvement of adjacent vertebral bodies, IVDS narrowing, periosteal proliferation, and/or spondylosis [1,3,15]. The osseous CT abnormalities identified in the present study were broadly consistent with those previously reported in the veterinary literature [3]. Vertebral endplate osteolysis was observed in 90.6% of dogs, comparable to the high prevalence of endplate involvement reported by Gomes et al. (87.3%), while periosteal proliferation and/or sclerosis was also commonly observed in both studies. However, Gomes et al. retrospectively evaluated non-contrast-enhanced CT examinations and focused primarily on the characterization of osseous abnormalities. Although the epidural space and paraspinal tissues were also assessed, no presumed epidural empyema or paraspinal fluid collection/abscess was identified. In our study, postcontrast CT revealed paravertebral soft tissue abnormalities in 87.5% of dogs and presumptive spinal epidural empyema in 53.1%. The high prevalence of these findings, together with the significant association between their evolution and clinical outcome, suggests that postcontrast assessment of paravertebral and epidural soft tissues provides clinically relevant information and should be included in the CT evaluation of dogs with suspected discospondylitis.

Among the soft tissue abnormalities identified in the present study, presumptive SEE was particularly common, affecting 53.1% of dogs, compared with the 3.9% prevalence previously reported in dogs [1]. SEE represents an accumulation of purulent material within the epidural space [23,24] and, in human patients, most commonly develops through local extension of vertebral osteomyelitis or discitis [25]. Although its pathogenesis is less clearly established in dogs, SEE has been reported in association with discospondylitis and may contribute to neurological dysfunction through direct neural compression and/or ischemic injury secondary to thrombosis or vasculitis [26,27]. The comparatively high prevalence in our population should, however, be interpreted cautiously. CT findings considered suggestive of SEE are not specific and may overlap with epidural phlegmon, granulomatous tissue, hematoma, or neoplastic infiltration [7]. Consequently, some lesions classified as presumptive SEE in the present study may have represented other forms of epidural inflammatory tissue, potentially contributing to the higher prevalence observed. MRI, with its superior soft tissue characterization, would have been valuable for further characterization and confirmation of these epidural abnormalities [26].

In contrast to these soft tissue abnormalities, interpretation of osseous changes during follow-up may be more complex because imaging and clinical recovery do not necessarily occur simultaneously. A previous study considered increasing sclerosis and osseous bridging as radiographic features of recovery, reflecting new bone formation, whereas progressive osteolysis was interpreted as disease progression [10]. That study demonstrated a temporal discrepancy between clinical and radiographic recovery, with osteolysis continuing to progress despite early clinical improvement and reparative changes becoming apparent only later during follow-up. This temporal lag may partly explain why changes in osteolysis and IVDS narrowing were not significantly associated with clinical outcome in our study, as persistent or progressive osseous destruction may not necessarily indicate an unfavorable clinical response at a given follow-up time point. Increasing sclerosis may nevertheless represent reparative bone formation, as experimental CT studies have shown that increased bone attenuation is associated with histological evidence of ossification during bone healing [28]. However, sclerosis is also a recognized CT feature of canine discospondylitis at presentation [3] and may coexist with active osteolytic changes. Therefore, although increasing sclerosis may be consistent with bone healing, its evolution was not considered sufficiently specific to be classified unequivocally as favorable or unfavorable and was not incorporated into the CT Evolution Score. Only 5 dogs in the present study underwent more than one follow-up CT examination, limiting our ability to characterize the temporal evolution of these osseous changes. Prospective longitudinal studies involving larger populations and serial CT examinations at standardized time points are warranted to determine whether a temporal lag between clinical improvement and CT evidence of osseous recovery, similar to that previously described radiographically [10], also occurs on CT.

Six dogs (6/32, 18.8%) underwent surgical treatment in addition to medical management. The limited number of surgically treated dogs precluded a meaningful statistical comparison between medically and surgically managed dogs. Nevertheless, surgical treatment should be considered when interpreting CT evolution, as surgical intervention and the underlying indications for surgery may have influenced both clinical and imaging outcomes. Favorable CT evolution following surgical management has also been reported in a dog with discospondylitis treated with external stabilization, in which sequential CT examinations documented the successful outcome [22]. Similarly, previous studies have included surgically treated dogs that subsequently showed favorable clinical evolution [1,11,15]. Therefore, the inclusion of surgically treated dogs allowed consideration of the whole spectrum of the population that should be included when interpreting the relationship between CT evolution and clinical outcome in the present study. Larger studies are needed to determine whether the pattern or timing of CT changes differs between medically and surgically managed dogs.

The potential clinical value of CT for monitoring response to treatment should also be considered in relation to previous studies evaluating radiographic and MRI follow-up of canine discospondylitis. Radiographic evolution may lag behind clinical recovery, particularly in mature dogs, in which progressive osteolysis may persist for several weeks despite successful treatment [10]. Consequently, radiographic findings alone were considered unreliable for assessing early response to treatment, and clinical improvement remained the primary indicator of therapeutic efficacy [10]. MRI may demonstrate abnormalities similar to those identified on CT and is considered more sensitive for the early detection of discospondylitis in veterinary patients [1,3,4,5,29]. However, despite its high diagnostic sensitivity, the clinical value of MRI for monitoring treatment response remains uncertain. In a previous follow-up study, no individual MRI feature was significantly associated with clinical resolution, and the subjective MRI classification of active versus inactive disease showed low sensitivity for clinical status [11]. Accordingly, routine follow-up MRI was not considered clinically useful in dogs in which clinical signs had resolved. In contrast, our study identified significantly more favorable evolution of paravertebral soft tissue and epidural abnormalities in dogs with a good clinical outcome, while the CT Evolution Score was significantly associated with clinical outcome. These findings suggest that CT may provide clinically relevant information regarding treatment response by combining assessment of osseous remodeling with postcontrast evaluation of inflammatory soft tissue abnormalities. CT may also offer practical advantages over MRI in veterinary practice, including greater availability and lower cost. However, because CT involves exposure to ionizing radiation, follow-up examinations should be justified on a case-by-case basis in accordance with the ALARA principle. Its clinical value may therefore be greatest in dogs showing incomplete or unfavorable clinical improvement, in which persistent or progressive disease is suspected, and CT findings may help guide further management. However, the present study evaluated associations between CT evolution and clinical outcome and was not designed to determine whether follow-up CT influences clinical decision-making or improves patient outcomes. Favorable CT changes should not be interpreted as establishing treatment success.

A similar approach has been advocated in human medicine. Although MRI is generally considered the imaging modality of choice for the diagnosis of discospondylitis, particularly for the detection of associated epidural or paravertebral abscesses, CT has also been proposed as a useful modality for assessing treatment response [12,13,14]. However, these studies have emphasized the limited correlation between follow-up imaging and clinical recovery. Persistent or even worsening imaging abnormalities may be observed despite clinical and biological improvement and do not necessarily indicate treatment failure or justify prolonged antimicrobial therapy [13,14]. Interestingly, soft tissue abnormalities appear to resolve more consistently during recovery than osseous abnormalities, with a significant reduction in soft tissue infiltration reported on follow-up CT and MRI, whereas other imaging abnormalities showed less consistent evolution [14]. This observation parallels the findings of the present study, in which improvement in paravertebral soft tissue and epidural abnormalities was associated with a good clinical outcome, while changes in osteolysis, spondylosis, or IVDS narrowing were not. Consequently, routine follow-up imaging is generally not advocated in human patients showing favorable clinical and biological responses, whereas additional imaging may be warranted when treatment failure or neurological deterioration is suspected [13,14]. A more selective approach has also been proposed, combining clinical status and inflammatory biomarkers as initial indicators of treatment response and reserving follow-up imaging for patients considered at greater risk of treatment failure [6]. A similar strategy may be relevant in dogs with discospondylitis, with clinical evolution and inflammatory biomarkers guiding the need for additional CT assessment rather than systematic imaging during follow-up.

CRP is a sensitive, although nonspecific, biomarker of discospondylitis in dogs [30,31], and its decrease during treatment may provide an additional indicator of response. In human patients with discospondylitis, CRP is similarly considered useful for monitoring treatment response [32], and changes in CRP have been reported to correlate more closely with soft tissue than with osseous changes on follow-up MRI [33]. In the present study, changes in soft tissue and epidural abnormalities were associated with clinical outcome, whereas changes in osseous abnormalities were not, although the relationship between CRP and individual CT features was not directly assessed. Given the limited and non-systematic availability of follow-up CRP measurements, CRP evolution was considered descriptively only, as the small number of evaluable cases did not allow robust assessment of its association with CT evolution or clinical outcome. Among the eight dogs in which CRP normalized, seven had both a positive CT Evolution Score and a good clinical outcome. However, this descriptive observation should be interpreted cautiously given the small number of dogs with available follow-up CRP measurements. Taken together with previous reports, clinical evolution and CRP may therefore provide complementary information for monitoring dogs with discospondylitis and help identify cases with an incomplete or unfavorable response in which follow-up CT may provide additional clinically relevant information.

The main limitations of this study are inherent to its retrospective design. The single-center setting and relatively small study population may have introduced selection bias, limited the statistical power of the analyses, and reduced the generalizability of the findings to the broader population of dogs with discospondylitis. In addition, requiring at least one follow-up CT examination as an inclusion criterion introduced a specific selection bias, as dogs that died or were euthanized before follow-up imaging could not be included. This may have resulted in an underrepresentation of dogs with poor outcomes and should be considered when interpreting the observed association between CT Evolution Score and clinical outcome. The retrospective review also resulted in variable availability of clinical, laboratory, and microbiological data. Microbiological sampling was not standardized and varied according to the individual clinical context and attending clinician’s discretion. In particular, the choice between CT- and US-guided disc fine-needle aspiration was based on the accessibility of the affected disc and the radiologist’s judgment regarding the most appropriate sampling approach. The limited sample size also reduced the robustness of the ROC analysis and derived cut-off. In dogs with multifocal discospondylitis, CT changes across affected IVDS were averaged to maintain the dog as the statistical unit and avoid non-independence of observations. However, this approach may have obscured discordant evolution between individual sites and limited the ability to detect associations with clinical outcome. In addition, clinical outcome was determined from information available in the medical records rather than using a standardized prospective clinical assessment, which may have introduced some degree of outcome misclassification.

The diagnosis of discospondylitis remained presumptive, as definitive confirmation was not available in most dogs. Positive bacterial cultures from direct IVD samples were obtained in 10 dogs, while two additional dogs had cytological evidence of inflammation despite negative bacterial culture. No histopathological examination was performed. Therefore, although all included dogs had clinical and CT findings considered consistent with discospondylitis, the possibility that lesions of different underlying etiologies were included cannot be completely excluded. Similarly, epidural abnormalities classified as presumptive SEE were not systematically confirmed by MRI, surgery, or histopathology and may therefore have represented other forms of epidural inflammatory tissue. CT examinations were performed as part of routine clinical care, and acquisition protocols and intervals between initial and follow-up examinations were not standardized. Consequently, dogs were evaluated at different stages of treatment and disease evolution, which may have influenced the CT changes observed. Furthermore, only 5 dogs underwent more than one follow-up CT examination, precluding detailed assessment of the temporal evolution of CT abnormalities and of a potential time lag between clinical and imaging recovery. Treatment also differed between dogs, including different antimicrobial protocols and surgical intervention in some dogs, which may have influenced clinical and CT evolution and could not be controlled for retrospectively.

Finally, the CT Evolution Score proposed in this study should be considered exploratory. The score was developed and evaluated within the same relatively small cohort, without external validation, and the limited number of dogs with poor outcomes may have reduced the stability and precision of the logistic regression estimates, discriminatory performance, and ROC-derived cut-off, which should therefore be interpreted cautiously. The reported discriminatory performance and ROC-derived cut-off may therefore be sample-dependent. In addition, the equal weighting assigned to individual CT features was chosen for simplicity in the absence of established feature-specific weighting and has not been independently validated. Accordingly, the proposed cut-off should not be interpreted as a validated threshold for clinical decision-making. Larger prospective, multicenter studies using standardized treatment, clinical assessment, and serial imaging protocols are warranted to externally validate the CT Evolution Score before its potential clinical application.

## 5. Conclusions

In conclusion, this study highlights the evolution of CT findings observed during follow-up of dogs with suspected discospondylitis and their relationship with clinical outcome. Improvement in paravertebral soft tissue and epidural abnormalities was associated with a good clinical outcome, whereas changes in osseous abnormalities were less closely related to clinical evolution. The CT Evolution Score developed in this study was significantly associated with clinical outcome, showed good discriminatory ability (AUC = 0.87) and demonstrated moderate interobserver reliability (ICC = 0.72), suggesting the potential value of assessing CT changes collectively rather than relying on individual imaging features. However, this score should be considered exploratory, as it was developed and evaluated within the same relatively small cohort and has not been externally validated. Follow-up CT may therefore provide complementary information in dogs with incomplete or unfavorable clinical improvement, alongside clinical assessment and inflammatory biomarkers. However, the present findings do not establish that CT improves clinical decision-making or patient outcomes. Further multicenter prospective studies with larger populations and standardized serial CT examinations are needed to validate these findings and determine the optimal role and timing of CT in the follow-up of canine discospondylitis.

## Figures and Tables

**Figure 1:**
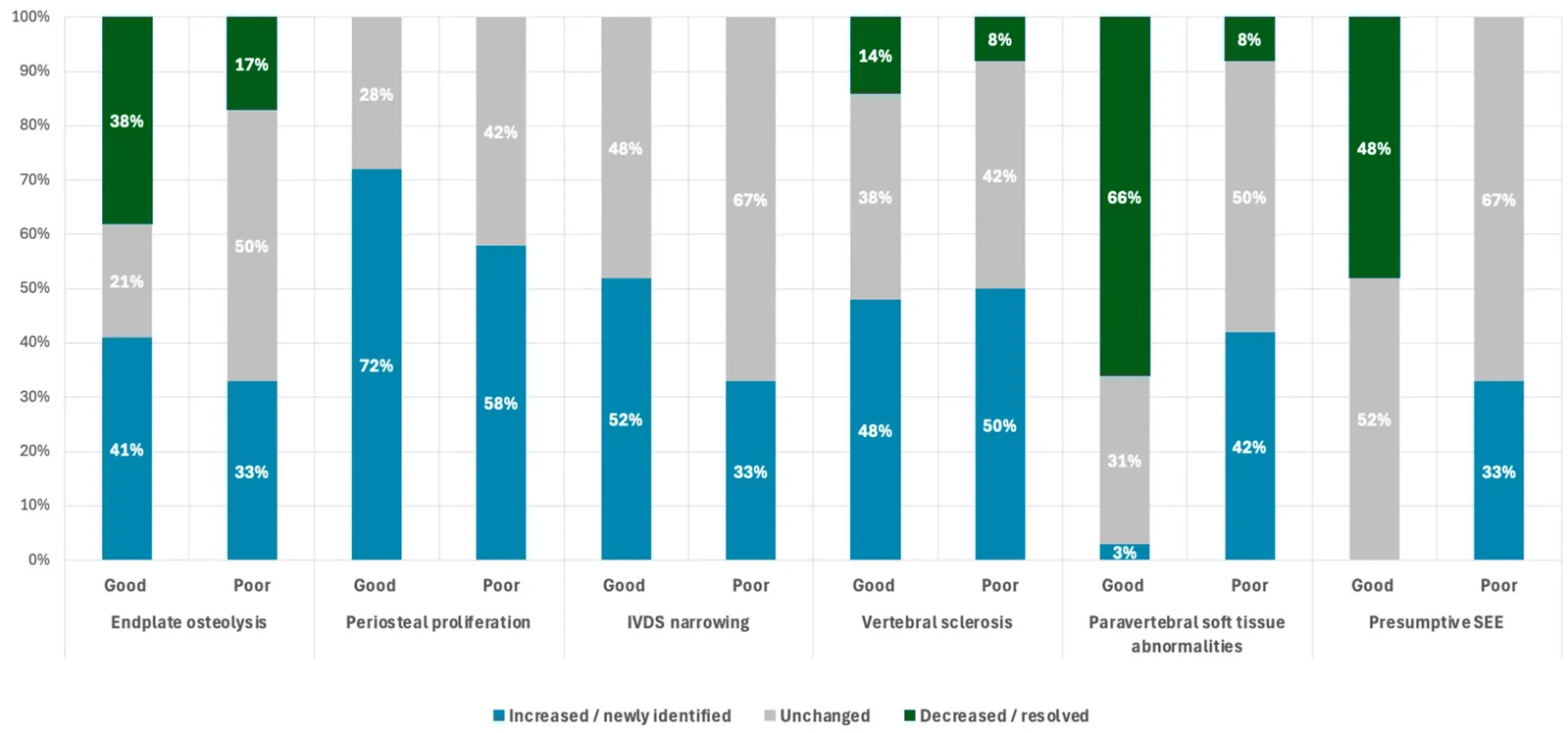
Evolution of individual CT findings from the initial to the first follow-up CT examination according to clinical outcome. Each affected intervertebral disc space was evaluated individually. IVDS, intervertebral disc space; SEE, spinal epidural empyema.

**Figure 2:**
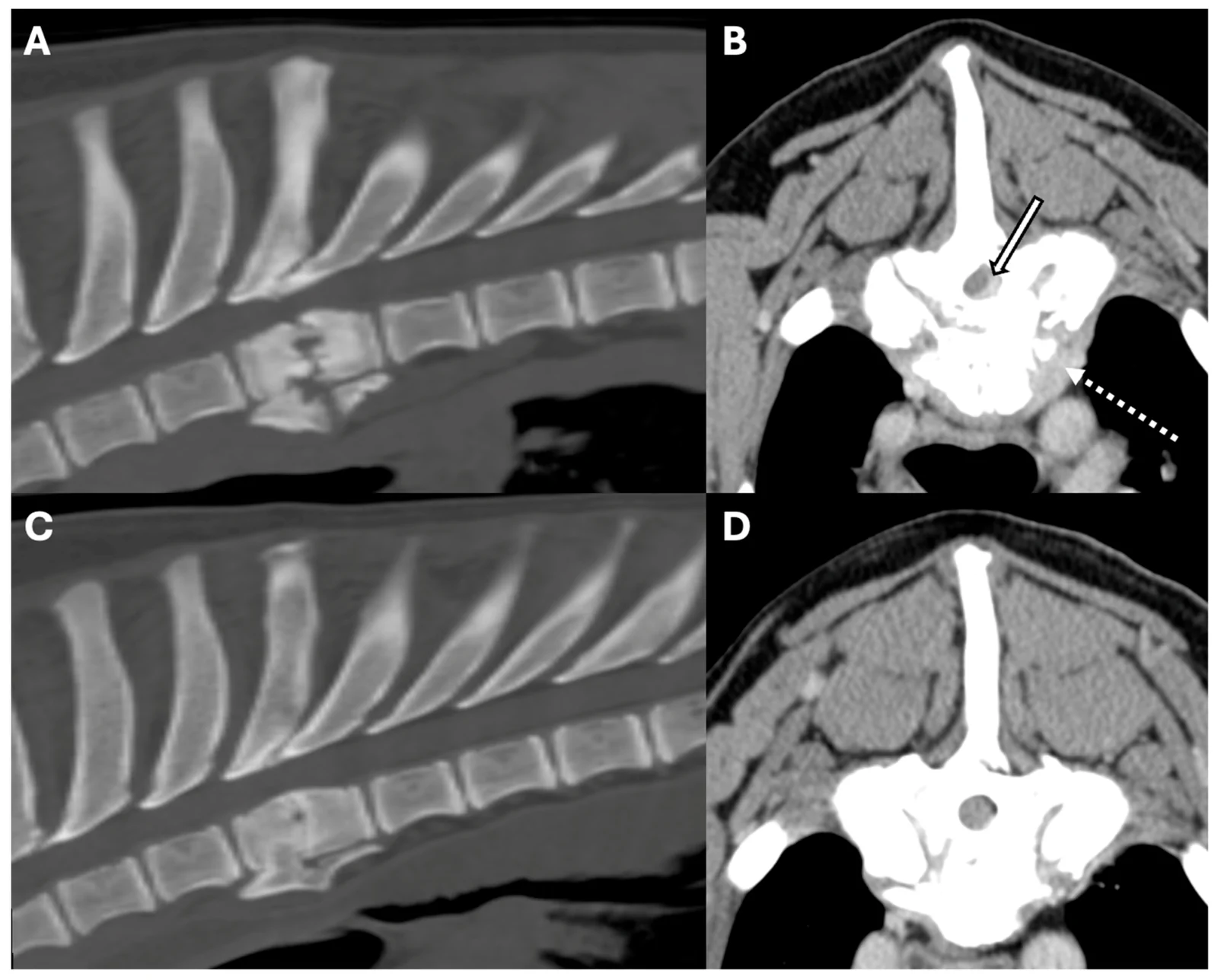
CT images of T5–T6 discospondylitis in an 8-month-old spayed female Beauceron at initial presentation (**A**,**B**) and 2-month follow-up (**C**,**D**). (**A**) Sagittal reconstruction in a bone window showing narrowing of the T5–T6 intervertebral disc space associated with severe osteolysis of the adjacent vertebral endplates and peripheral periosteal proliferations. (**B**) Transverse reconstruction at the level of the T5–T6 intervertebral disc space in a soft tissue window following intravenous administration of iodinated contrast medium, showing heterogeneously contrast-enhancing thickening of the ventral paravertebral soft tissues (dotted arrow) and presumed epidural empyema (thick arrow). (**C**) Sagittal reconstruction in a bone window at 2-month follow-up showing decreased osteolysis, with smooth vertebral endplates, further narrowing of the T5–T6 intervertebral disc space and complete osseous bridging across the affected disc space. (**D**) Transverse postcontrast reconstruction in a soft tissue window at the same follow-up examination showing resolution of the paravertebral soft tissue abnormalities and presumed epidural empyema. The dog had a good clinical outcome, with a resolution of clinical signs at follow-up, and a positive CT Evolution Score.

**Figure 3:**
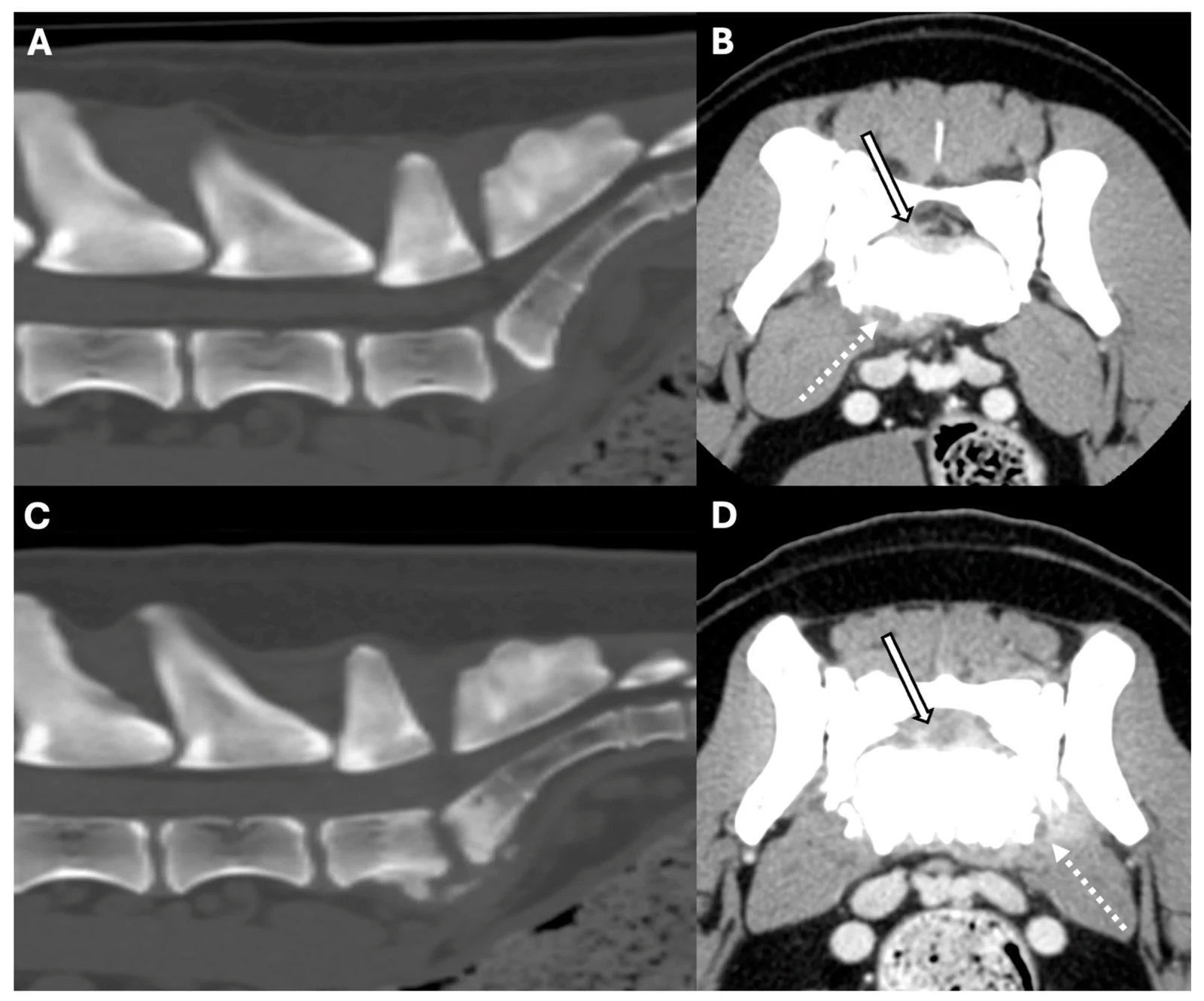
CT images of L7–S1 discospondylitis in a 3-year-old intact female Beauceron at initial presentation (**A**,**B**) and 2-month follow-up (**C**,**D**). (**A**) Sagittal reconstruction in a bone window showing smooth vertebral endplates and mild sclerosis of the cranial endplate of S1. (**B**) Transverse reconstruction at the level of the L7–S1 intervertebral disc space in a soft tissue window following intravenous administration of iodinated contrast medium, showing heterogeneously contrast-enhancing thickening of the ventral paravertebral soft tissues (dotted arrow) and presumed epidural empyema (thick arrow). (**C**) Sagittal reconstruction in a bone window at 2-month follow-up showing mild narrowing of the L7–S1 intervertebral disc space, development of vertebral endplate osteolysis, and increased ventral periosteal proliferation and sclerosis. (**D**) Transverse postcontrast reconstruction in a soft tissue window at the same follow-up examination showing a mild increase in the paravertebral soft tissue abnormalities (dotted arrow) and marked progression of the presumed epidural empyema (thick arrow), occupying most of the vertebral canal cross-sectional area. The dog had a poor clinical outcome, with persistent back pain, and a negative CT Evolution Score.

**Table 1:** CT Evolution Scoring System for Assessment of Discospondylitis Lesion Evolution.

CT Features	Decreased	Unchanged	Increased, Newly Identified
Osteolysis	+1	0	−1
IVDS narrowing	−1	0	+1
Periosteal proliferations	−1	0	+1
Paravertebral soft tissue abnormalities	+1	0	−1
Epidural soft tissue abnormalities	+1	0	−1
**Additional CT features**	**Present**		**Absent**
Complete bridging	+1		0
New discospondylitis site	−1		0

**Table 2:** Demographic and clinical characteristics of the 32 dogs with presumed discospondylitis included in the study.

**Breeds (n)**	Boxer	**3**
	German Shepherd	3
	Beauceron	2
	Tervueren	2
	Brittany Spaniel	2
	Cane Corso	2
	Labrador Retriever	2
	Newfoundland	2
	Staffordshire Bull Terrier	2
	Crossbreed	2
	American Bulldog	1
	American Bully	1
	Chowchow	1
	Doberman	1
	English Bulldog	1
	French Bulldog	1
	Jack Russel Terrier	1
	Malinois	1
	Pyrenean Mountain Dog	1
	Weimaraner	1
**Age (months) [median (range)]**		50 (8–129)
**Weight (kg) [median (range)]**		33 (8–65)
**Sex (n)**	Entire male	16
	Castrated male	6
	Entire female	4
	Spayed female	6
**Clinical signs at admission (n)**	Spinal hyperesthesia	21
	Additional neurological signs	13
	Pyrexia (>39.2 °C)	7
**Duration of clinical signs (n)**	Acute (<7 days)	7
	Subacute (7–30 days)	18
	Chronic (>30 days)	7

n: number of dogs.

**Table 3:** Microbiological results according to sampling site in dogs with presumed discospondylitis.

Sampling Method		Identified Pathogens (n)
**Percutaneous disc aspiration (CT- or US-guided)**		*Staphylococcus pseudintermedius* (3) ^†^
		*Staphylococcus* spp. (1)
		*Staphylococcus* coagulase + (1)
		*Escherichia coli* (1)
		*Klebsiella pneumoniae* (1) *
		*Pseudomonas aeruginosa* (1)
		*Serratia marcescens* (1)
**Intraoperatively sampling**		*Staphylococcus* spp. (1)
		*Streptococcus mitis* (1)
**Urine culture**		*Staphylococcus pseudintermedius* (3) ^†^
		*Staphylococcus* spp. (1)
		*Enterobacter cloacae* (1)
**Other sampling methods**	Articular joint aspirate	*Enterobacter cloacae* (1)
	Removed surgical material	*Klebsiella pneumoniae* (1) *
	Prostate aspirate	Negative

n: number of dogs. * Concordant IVD and surgical material culture results in one dog. ^†^ Concordant IVD and urine culture results in one dog.

**Table 4:** Associations between changes in CT findings and clinical outcome at first follow-up in the 32 dogs with suspected discospondylitis.

Variable	Statistical Test	Effect Estimate	*p*-Value
**Paravertebral soft tissue thickening**	Mann–Whitney U	Rank–biserial correlation = −0.882	<0.001
**Presumed empyema**	Mann–Whitney U	Rank–biserial correlation = −0.822	0.002
**Endplates osteolysis**	Mann–Whitney U	Rank–biserial correlation = −0.193	0.493
**Paravertebral proliferations/spondylosis**	Mann–Whitney U	Rank–biserial correlation = 0.096	0.689
**IVDS narrowing**	Mann–Whitney U	Rank–biserial correlation = −0.126	0.641
**CT Evolution Score**	Mann–Whitney U	Rank–biserial correlation = −0.733	0.01
	Binary logistic regression	OR, 0.40; 95% CI, 0.17–0.91	0.029

**Table 5:** Interobserver agreement for individual CT features and the CT Evolution Score.

CT Feature/Score	Agreement	Statistic	Interpretation
**Endplates osteolysis**	66%	κ = 0.530	Moderate
**Paravertebral proliferations/spondylosis**	78%	κ = 0.528	Moderate
**IVDS narrowing**	68%	κ = 0.382	Fair *
**Sclerosis**	72%	κ = 0.518	Moderate
**Paravertebral soft tissue thickening**	66%	κ = 0.457	Moderate
**Presumed empyema**	82%	κ = 0.701	Substantial
**CT Evolution Score**	-	ICC [2, 1] = 0.721 (95% CI, 0.557–0.831)	Moderate

* Low-prevalence categories may result in lower Cohen’s kappa values despite observed agreement. For IVDS narrowing, Gwet’s AC2 was 0.728 (95% CI, 0.606–0.849).

## Data Availability

The data presented in this study are available on request from the corresponding author, because of the privacy of the medical records of client-owned animals.

## Abbreviations

The following abbreviations are used in this manuscript:

IVD	intervertebral disc
CT	computed tomography
MRI	magnetic resonance imaging
IVDS	intervertebral disc space
MFS	modified Frankel Scale
CRP	C-reactive protein
DICOM	Digital Imaging and Communications in Medicine
PACS	Picture Archiving and Communication System
SEE	spinal epidural empyema
ICC	intraclass correlation coefficient
AC2	Gwet’s agreement coefficient 2
OR	odds ratio
CI	confidence interval
ROC	receiver operating characteristic
AUC	area under the curve
DPO	Double Pelvic Osteotomy
ALARA	as low as reasonably achievable
